# A 2-year multicenter, observational, prospective, cohort study on extracorporeal CO_2_ removal in a large metropolis area

**DOI:** 10.1186/s40560-019-0399-8

**Published:** 2019-08-20

**Authors:** J. L. Augy, N. Aissaoui, C. Richard, E. Maury, M. Fartoukh, A. Mekontso-Dessap, R. Paulet, N. Anguel, C. Blayau, Y. Cohen, J. D. Chiche, S. Gaudry, S. Voicu, A. Demoule, A. Combes, B. Megarbane, E. Charpentier, S. Haghighat, M. Panczer, J. L. Diehl

**Affiliations:** 1 Service de Médecine Intensive Réanimation, AP-HP, Hôpital Européen Georges Pompidou, Paris, France; 2 Service de Médecine Intensive Réanimation, AP-HP, Hôpital de Bicètre, Le Kremlin Bicètre, France; 3 Service de Médecine Intensive Réanimation, AP-HP, Hôpital Saint-Antoine, Paris, France; 4 Service de Réanimation Polyvalente, AP-HP, Hôpital Tenon, Paris, France; 5 Service de Médecine Intensive Réanimation, AP-HP, Hôpital Henri Mondor, Créteil, France; 6 Service de Réanimation Polyvalente, Centre Hospitalier de Longjumeau, Longjumeau, France; 7 Service de Réanimation Polyvalente, AP-HP, Hôpital Avicenne, Bobigny, France; 8 Service de Médecine Intensive Réanimation, AP-HP, Hôpital Cochin, Paris, France; 9 Service de Réanimation Polyvalente, AP-HP, Hôpital Louis Mourier, Colombes, France; 10 Service de Médecine Intensive Réanimation, AP-HP, Hôpital Lariboisière, Paris, France; 11 AP-HP, Groupe Hospitalier Pitié-Salpêtrière Charles Foix, Service de Pneumologie, Médecine Intensive et Réanimation, Département R3S, Sorbonne Université, INSERM, UMRS1158 Neurophysiologie Respiratoire Expérimentale et Clinique, Paris, France; 12 Service de Médecine Intensive Réanimation, AP-HP, Hôpital Pitié-Salpétrière, Institut de Cardiologie, Paris, France; 13 AP-HP, Office du Transfert de Technologie et des Partenariats Industriels, Paris, France; 14 AP-HP, Agence Générale des Equipements et des Produits de Santé, Paris, France; 15 Faculty of Pharmacy, INSERM UMR-S1140, Paris Descartes University, Paris, France

**Keywords:** Extracorporeal CO_2_ removal, Acute respiratory distress syndrome, COPD exacerbation, Safety

## Abstract

**Background:**

Extracorporeal carbon dioxide removal (ECCO_2_R) is a promising technique for the management of acute respiratory failure, but with a limited level of evidence to support its use outside clinical trials and/or data collection initiatives. We report a collaborative initiative in a large metropolis.

**Methods:**

To assess on a structural basis the rate of utilization as well as efficacy and safety parameters of 2 ECCO_2_R devices in 10 intensive care units (ICU) during a 2-year period.

**Results:**

Seventy patients were recruited in 10 voluntary and specifically trained centers. The median utilization rate was 0.19 patient/month/center (min 0.04; max 1.20). ECCO_2_R was started under invasive mechanical ventilation (IMV) in 59 patients and non-invasive ventilation in 11 patients. The Hemolung Respiratory Assist System (Alung) was used in 53 patients and the iLA Activve iLA kit (Xenios Novalung) in 17 patients. Main indications were ultraprotective ventilation for ARDS patients (*n* = 24), shortening the duration of IMV in COPD patients (*n* = 21), preventing intubation in COPD patients (*n* = 9), and controlling hypercapnia and dynamic hyperinflation in mechanically ventilated patients with severe acute asthma (*n* = 6). A reduction in median *V*_T_ was observed in ARDS patients from 5.9 to 4.1 ml/kg (*p* <0.001). A reduction in PaCO_2_ values was observed in AE-COPD patients from 67.5 to 51 mmHg (*p*< 0.001). Median duration of ECCO_2_R was 5 days (IQR 3–8). Reasons for ECCO_2_R discontinuation were improvement (*n* = 33), ECCO_2_R-related complications (*n* = 18), limitation of life-sustaining therapies or measures decision (*n* = 10), and death (*n* = 9). Main adverse events were hemolysis (*n* = 21), bleeding (*n* = 17), and lung membrane clotting (*n* = 11), with different profiles between the devices. Thirty-five deaths occurred during the ICU stay, 3 of which being ECCO_2_R-related.

**Conclusions:**

Based on a registry, we report a low rate of ECCO_2_R device utilization, mainly in severe COPD and ARDS patients. Physiological efficacy was confirmed in these two populations. We confirmed safety concerns such as hemolysis, bleeding, and thrombosis, with different profiles between the devices. Such results could help to design future studies aiming to enhance safety, to demonstrate a still-lacking strong clinical benefit of ECCO_2_R, and to guide the choice between different devices.

**Trial registration:**

ClinicalTrials.gov: Identifier: NCT02965079 retrospectively registered https://clinicaltrials.gov/ct2/show/NCT02965079

## Introduction

Extracorporeal CO_2_ removal (ECCO_2_R) is potentially a major therapeutic breakthrough in critical care [1, 2]. The two main conditions that could benefit from this technique are acute respiratory distress syndrome (ARDS) and very severe acute exacerbations of chronic obstructive pulmonary disease (AE-COPD). The main objective of ECCO_2_R in ARDS is to implement an ultra-protective invasive mechanical ventilation (IMV) strategy, mainly by decreasing the tidal volume (from the usually recommended value of 6 ml/kg (predicted body weight) to a value of 3–4 ml/kg) [3–8], and including as complementary options a rise in positive end-expiratory pressure (PEEP) as well as a diminution in respiratory rate [9]. The goals in AE-COPD are to prevent tracheal intubation and to shorten IMV duration [10–14]. Corresponding physiological respiratory benefits have been demonstrated, at the price however of hemolytic, hemorrhagic, and thrombotic complications [1, 2, 15]. Awaiting the results of current or planned RCTs, it has been suggested to use ECCO_2_R within clinical trials and/or to contribute to data registries [1, 2, 15, 16].

Accordingly, within the great Paris area, the use of the ECCO_2_R as part of the current care was rigorously organized. As a result of a referral, a report (supported by a clinician’s interviews and by a systematic analysis of the literature) was released in June 2014 by an institutional Agency for Health Technology Assessment attached to the Assistance Publique–Hôpitaux de Paris (AP-HP) [17]. The main recommendations were to establish a working group led by clinicians able to give a scientific opinion on the appropriateness of the ECCO_2_R activity, to authorize the use of ECCO_2_R in selected voluntary centers, to organize a systematic recording of the activity on an individual basis, and to reassess periodically the ECCO_2_R activity (on the basis of the available literature and of results of records). The project was supported by the Office of Technology Transfer and Partnerships Industrialists of the AP-HP and by the Institutional Pharmacy Agency (AGEPS). Test markets were concluded by the AGEPS with industrial firms, with a strict follow-up of the orders. We here report our initial experience based on the first 70 patients included in the corresponding registry.

## Methods

### Ethical and regulatory aspects

The study was approved by the Ethical Committee of the French Intensive Care Society and by the “Comité consultatif sur le traitement de l’information en matière de recherche dans le domaine de la santé,” a governmental committee on the use of information in the health domain. The study was registered on ClinicalTrials.gov: Identifier: NCT02965079. A written information form was given and orally explained to patients or proxies, who had the possibility to decline the utilization of data.

### Patient population and general organization

Patients were prospectively recruited during a 2-year period in 10 voluntary centers in Paris and its suburb. Initial training on how to operate devices was provided by the firms to nurses and medical teams in each center before any utilization. New training sessions were regularly organized, as requested by medical teams. Clinicians were asked to fill a dedicated form for each ECCO_2_R patient and to strictly follow user’s guides developed by the firms.

### ECCO_2_R management

Two devices, Hemolung Respiratory Assist System (Alung Technologies, Pittsburgh, USA) and iLA Activve iLA kit (Xenios Novalung, Heilbronn, Germany), were used during the period. The vascular access was achieved by mean of a specific double-lumen 15.5-Fr veno-venous catheter (either right jugular or femoral site) for the Hemolung device and by mean of double-lumen 18 Fr (right jugular site) or 24 Fr (femoral site) for the iLa Activve system, using Novaport Twin (18, 22, or 24 Fr) catheters (Xenios Novalung, Heilbronn, Germany). Extracorporeal blood flow rates are generally comprised between 350 and 550 ml/min for the Hemolung system and between 500 ml/min and 1500 ml/min for the iLa Activve system. The sweep gas flow was adjusted for controlling hypercapnia, for achieving protective or ultraprotective ventilation, and for unloading the respiratory muscles depending on the indications for ECCO_2_R and on the clinical courses of the patients. All patients were treated by continuous intravenous infusion of unfractionated heparin and monitored by serial measurements of anti-Xa activity, with a therapeutic range between 0.3 and 0.6 IU/ml.

### Data collection

The individual dedicated form included the following information: baseline characteristics, indication for ECCO_2_R, type of ECCO_2_R device, type and site of veno-venous ECCO_2_R catheter, type of ventilatory support, concomitant treatments, adverse events (AE) and serious adverse events (SAE), reason for ECCO_2_R discontinuation, and respiratory and general follow-up until ICU discharge or death.

### Endpoints

The primary outcome was the number of patients treated by ECCO_2_R per month and per center during the 2-year study period. Based mainly on the annual number of ARDS and AE-COPD admissions, a recruitment of 200 patients (100 patients per year) was roughly anticipated. Secondary endpoints were related to ECCO_2_R physiological efficacy (based mainly on respiratory assessment after 24 h of use) and safety, length of mechanical ventilation, and ICU and hospital survival. Main safety endpoints were defined as follows: bleeding deemed as clinically significant by clinicians; biological hemolysis defined by a serum-free hemoglobin level higher than 100 mg/l; clinical hemolysis when associated to jaundice, hemoglobinuria, or impaired renal function; thrombosis, membrane clotting, and catheter infection. Clinically significant bleeding was defined by the need of RBC transfusion, whatever the number of RBC units, and/or need to stop continuous intravenous unfractionated heparin infusion, and/or need of surgery or any interventional procedure to control bleeding, and/or association to hemodynamic instability. Membrane clotting was defined as apparent membrane clotting after a daily visual inspection or suspected massive membrane clotting leading to ECCO_2_R cessation and further confirmed by analysis of the circuit.

### Statistical analyses

The primary outcome is reported as median and extreme values. Other continuous variables are reported as median (interquartile range) (IQR) and categorical variables are reported as count and proportion. Categorical variables were compared using the Fisher exact test. Between-group comparisons of continuous variables were performed using the chi-square test. All analyses were made on R software (R version 3.3.2). All *p* values less than 0.05 were considered significant.

## Results

Seventy patients were treated by ECCO_2_R during the 2-year study period. Median monthly utilization rate by center was 0.19 patients (min 0.04; max 1.2). During the period, the median ICU admission rates of ARDS and AE-COPD patients were 8.16 (min 5.00; max 9.91) and 2.19 (min 1.50; max 6.79), respectively. Fifty-three patients were treated with the Hemolung device and 17 with the iLA Activve device.

Baseline demographic characteristics of the patients are reported in Table 1. The severity of patients at ICU admission was assessed by a median SAPS II of 43 (35–45). Main indications for ECCO_2_R were ultraprotective ventilation in 24 (34%) ARDS patients; shortening the duration of IMV in 21 (30%) COPD patients; preventing intubation in 9 (13%) COPD patients who failed non-invasive ventilation (NIV); and controlling hypercapnia and dynamic hyperinflation in mechanically ventilated patients with severe acute asthma (*n* = 6; 9%). Etiology of ARDS was pneumonia in 19 patients, acute exacerbation of interstitial lung diseases in 3, lung toxicity of chemotherapy in 1, and smoke inhalation in 1. Table 2 indicates baseline demographics in the two main indications: ARDS and AE-COPD. Other 10 indications were acute exacerbation of interstitial lung disease without ARDS criteria (*n* = 2), bronchiolitis (*n* = 2), bridge to lung transplantation, post-extubation laryngeal edema, unilateral pneumonia, malignant tracheal obstruction, acute exacerbation of chronic restrictive pulmonary disease, and difficult IMV weaning outside chronic respiratory insufficiency (*n* = 1 each). Nineteen patients (11 ARDS and 8 AE-COPD) were treated by ECCO_2_R as part of a registered interventional clinical trial.

**Table 1:** Baseline characteristics of the population

Age (years)	65 (61–74)
Male patients	41 (59%)
BMI (kg/m^2^)	25.3 (22.0–32.3)
Comorbidities	
Chronic respiratory disease	30 (43%)
Chronic cardiac disease	11 (16%)
Chronic kidney disease	8 (11%)
Diabetes	12 (17%)
SAPS II	43 (35–45)
pH	7.28 (7.22–7.32)
PaCO_2_ (mmHg)	64 (56–73)
*V*_T_ (ml/kg PBW)	6.2 (5.9–7.9)
Respiratory setting	
IMV	59 (84%)
NIV	11 (16%)
Concomitant treatments	
Vasopressors	27 (39%)
Renal replacement therapy	8 (11%)
Neuromuscular blockade	37 (53%)
Steroids	16 (23%)
Prone positioning	4 (6%)

Results are expressed as median (IQR) for continuous variables and count and proportion for categorical variables

*Abbreviations*: *BMI* body mass index, *SAPS* Simplified Acute Physiology Score, *V*_T_ tidal volume, *PBW* predicted body weight, *IMV* invasive mechanical ventilation, *NIV* non-invasive ventilation

**Table 2:** Demographics, ventilator course, and clinical course according to the two main indications of ECCO_2_R

	AE-COPD (*n* = 30)	ARDS (*n* = 24)
Age (years)	66 (61–72)	66 (63–77)
Male: *n* (%)	12 (40%)	17 (71%)
FEV1 (L)	0.97 (0.69–1.2)	NA
FEV1 (%)	35% (29.5–53)	NA
PaO_2_/FiO_2_ (mmHg)	NA	131 (100–190)
SAPS II:	36 (32–50)	48 (43–62)
Use of vasopressors: *n* (%)	10 (33%)	14 (58%)
Type of ventilatory support		
IMV: *n* (%)	21 (70%)	24 (100%)
NIV: *n* (%)	9 (30%)	NA
IMV settings before ECCO_2_R:		
*V*_T_ (ml/kg PBW)	8.0 (7.8–8.1)	5.9 (5.5–6.0)
Applied PEEP (cmH_2_O)	0 (0–0)	10 (5–15.5)
FiO_2_ (%)	35 (30–38)	60 (50–70)
Measured parameters under IMV before ECCO_2_R:		
Plateau pressure (cmH_2_O)	NA	28 (27–29)
Total PEEP (cmH_2_O)	9 (7–11)	NA
pH	7.30 (7.25–7.32)	7.24
PaCO_2_ (mmHg)	67.5 (60.75–73.25)	58.0 (48.0–65.0)
ECCO_2_R device		
iLa Activve: *n* (%)	5 (17%)	6 (25%)
Hemolung: *n* (%)	25 (83%)	18 (75%)
IMV settings and measured parameters at day 1 after starting ECCO_2_R:		
*V*_T_ (ml/kg PBW)	7.98 (7.70–8.10)	4.1 (3.9–4.8)*
pH	7.39 (7.26–7.42)*	7.31 (7.24–7.36)**
PaCO_2_ (mmHg)	51.0 (45.5–56.0)*	51.0 (44.5–55.7)
ECCO_2_R duration (days):	6.5 (3.25–8)	4 (2–6)
IMV duration (days)	10 (4.75–15.25)	8.5 (5.5–14.25)
Course of ventilator support		
IMV weaning success (IMV patients)	16 (76%)	7 (29%)
Intubation (NIV patients)	1 (11%)	NA
Mortality: *n* (%)	10 (31%)	17 (71%)
Linked to ECCO_2_R	2 (7%)	1 (4%)

Results are expressed as median (IQR) for continuous variables and count and proportion for categorical variables

*Abbreviations*: *FEV1* forced expiratory volume in 1 s, *BMI* body mass index, *SAPS* Simplified Acute Physiology Score, *IMV* invasive mechanical ventilation, *NIV* non-invasive ventilation, *V*_T_ tidal volume, *PBW* predicted body weight, *PEEP* positive end-expiratory pressure

*p < 0.01 as compared to values before ECCO_2_R

**p < 0.05 as compared to values before ECCO_2_R

The median time between ICU admission and ECCO_2_R initiation was 3 days [1–9]. For IMV patients, the median IMV duration before ECCO_2_R initiation was 2 days [1–5]. Table 3 indicates the technical settings and medical conditions in relation to the two medical devices.

**Table 3:** Technical settings and medical conditions according to the use of the two medical devices

	Hemolung *n* = 53	iLa Activve *n* = 17	*p*
Catheter size: *n* (%)			
15.5 F	53 (100)		
18 F		9 (53)	
24 F		8 (47)	
Canula site: *n* (%)			0.539
Right internal jugular vein	32 (60)	9 (53)	
Femoral vein	21 (40)	8 (47)	
Respiratory support: *n* (%)			0.895
IMV	44 (83)	15 (88)	
NIV	9 (9)	2 (6)	
ECCO_2_R duration (days)	5 (3–8)	5 (3–9)	0.812
Number of membrane lung per patient	1	1	1

Results are expressed as count and proportion for categorical variables

Table 2 indicates the changes in ventilator parameters at day 1 after starting ECCO_2_R in AE-COPD and ARDS patients. A significant reduction in median *V*_T_ was observed in ARDS patients from 5.9 to 4.1 ml/kg (predicted body weight, PBW), in line with an ultra-protective ventilation strategy. A significant reduction in PaCO_2_ values was observed in AE-COPD patients.

Median ECCO_2_R duration was 5 days [3–8] without difference between the two medical devices (*p* = 0.812). Main reasons for ECCO_2_R discontinuation were improvement in clinical condition in 33 patients, ECCO_2_R-related adverse events in 18 patients, limitation of life-sustaining therapies or measures decision in 10 patients, and death in 9 patients (Table 4). No patient among the 33 patients who weaned from ECCO_2_R because of improvement died in ICU. Twenty-eight patients among the 59 under IMV when starting ECCO_2_R were weaned from IMV. Among the 11 patients under NIV when starting ECCO_2_R, 1 needed to be intubated and 5 were successfully weaned from NIV. There was no transition to ECMO for any patient. Thirty-five patients died in the ICU. In-hospital mortality was 51.5% (36 deceased patients, 6 missing data mainly due to transfer to another institution). Three deaths were judged as ECCO_2_R-related by clinicians in charge (2 intra-cranial bleedings without heparin overdosing and 1 cardiac tamponade following a right jugular vein catheterization).

**Table 4:** Clinical course after ECCO2R initiation depending on the initial ventilatory support

Type of ventilatory support when starting ECCO_2_R	IMV	NIV
*n* (%)	59 (84%)	11 (16%)
Reasons for stopping ECCO_2_R		
Success	28 (47%)	5 (45%)
Adverse events	17 (29%)	1 (9%)
Transition to ECMO	0 (0%)	0 (0%)
Death	7 (12%)	2 (18%)
Limitation of life-sustaining therapy decision	7 (12%)	3 (27%)
IMV weaning	28 (47%)	NA
NIV weaning	NA	5 (45%)
Tracheal intubation	NA	1 (9%)
In-ICU mortality	27 (46%)	7 (64%)

Results are expressed as count and proportion for categorical variables

*Abbreviations*: *IMV* invasive mechanical ventilation, *NIV* non-invasive ventilation

At least one ECCO_2_R-related adverse event was reported in 38 patients (Table 5). Hemolysis was reported in 21 patients all treated with the Hemolung device and led to ECCO_2_R discontinuation in 6 patients. Lung membrane clotting was reported in 11 patients leading to ECCO_2_R discontinuation in 6. Clinically significant bleeding was reported in 17 patients, 7 of whom needed RBC transfusion and 3 needed specific treatments (catheter-selective embolization in 3, with a further need for surgery in 1). Bleeding was the reason for ECCO_2_R discontinuation in 6 patients.

**Table 5:** ECCO_2_R-related adverse events

*n* (%)	Hemolung *n* = 53	iLa Activve *n* = 17	*p*
Catheterization failure	2 (4)	1 (4)	1
Biological hemolysis	15 (28)	0 (0)	0.033
Clinically significant hemolysis	6 (11)	0 (0)	0.147
Bleeding	16 (30)	1 (6)	0.042
Membrane clotting	4 (8)	7 (41)	< 0.001
Catheter infection	0 (0)	1 (6)	0.075
Device malfunction	4 (8)	2 (12)	0.638
ECCO_2_R-related death	3 (6)	0 (0)	0.316

Results are expressed as counts and proportions. Biological hemolysis was defined by at least one measurement of serum-free hemoglobin higher than 100 mg/l without clinically significant hemolysis

Device malfunction leading to ECCO_2_R discontinuation was reported in 6 patients. Software was involved in 3 of the 4 Hemolung malfunctions, 1 malfunction being not investigated. Air in the circuit was reported in 2 patients treated with the iLa Activve device.

## Discussion

Our study describes a collaborative institutional multicenter process for implementation of new ECCO_2_R medical devices in a large metropolis area, with the aim to establish a registry, in accordance with national recommendations [16]. Despite a low rate of utilization, mainly in COPD and ARDS patients, we were able to report a confirmed physiological efficacy and different safety profiles between the devices.

Primary end-point was the rate of utilization during a first 2-year period. We report a low rate of use, predominantly in AE-COPD and ARDS patients, with a maximal value of 1.2 patients/month/center. Altogether, ECCO_2_R was used in less than 2% of ARDS and AE-COPD patients during the corresponding period. Such a result is in line with a previous national survey in 239 ICUs and probably illustrates the lack of formally demonstrated strong outcome benefit of such expensive medical devices [18]. Nevertheless, our study is one of the largest ECCO_2_R multicenter initiatives and permitted to confirm and/or to extend efficacy and safety information as well as to introduce ECCO_2_R as a therapeutic option in selected centers.

As expected, we report a preferential ECCO_2_R use in ARDS and AE-COPD patients. Contrary to other reports, we observed a higher number of AE-COPD patients as compared to ARDS patients, most of them being treated by ECCO_2_R while on IMV [6, 18]. Interestingly, we observed that ECCO_2_R was used, as the third main indication, in acute severe asthma patients while on IMV, despite the scarcity of data [19–21]. ECCO_2_R indication in asthma was probably driven by a strong physiopathological rational, aiming at both limiting the levels of hypercapnia and of dynamic hyperinflation.

The physiological efficacy of ECCO_2_R was confirmed by the respiratory parameters observed at day 1. A significant reduction in median *V*_T_ was observed in ARDS patients from 5.9 to 4.1 ml/kg (PBW); in line with an ultra-protective ventilation strategy. However, our results are limited by the lack of systematic recordings of respiratory rate and plateau pressure after initiation of ECCO_2_R in ARDS patients. As a consequence, we cannot precisely address these additional components of an ultraprotective ventilation strategy. A significant reduction in PaCO_2_ values was observed in AE-COPD patients, with no modification in median *V*_T_. No systematic assessment of dynamic hyperinflation and/or work of breathing was planned in the study and accordingly recorded in the dedicated form, and no specific recommendations were made for respiratory setting adjustments under ECCO_2_R, so we cannot indicate to what extent improvements in such important parameters were also observed in AE-COPD patients. The median ECCO_2_R duration was of 5 days whatever the device, which is less than the maximal duration of use indicated by the firms.

We observed a 50% ICU mortality rate. Such a high mortality rate could be explained by the inclusion of very severe patients (as illustrated by the number of limitation of life-sustaining therapies or measure decisions), by the inclusion of patients with very severe conditions outside the main ECCO_2_R indications (possibly of poorer prognosis), and by a learning curve in the majority of the centers, therefore possibly minimizing ECCO_2_R benefits. The number of limitation of life-sustaining therapies or measure decisions could be explained mainly by the inclusion of ARDS patients, in which such decisions are frequent during the course of the disease [22]. Another explanation for the observed high mortality rate could be the lack of inclusion of trauma patients in the ARDS group. Indeed, trauma is a condition generally associated with a better short-term prognosis than for other ARDS etiologies [23]. We observed 3 (4%) ECCO_2_R-related deaths, which seem higher than in previous reports [6, 8, 24] and higher than the treatment-related mortality hypothesis retained in a physiological precision medicine study, aiming to identify the best ARDS candidates for inclusion in a randomized trial on ultra-protective ventilation [25].

Bleeding was reported in 17 (24%) patients, more frequently associated with the Hemolung device, despite a similar heparin regimen. One explanation could be a more frequent occurrence of an acquired Willebrand disease, as recently suggested with the Hemolung device [26]. Further studies are needed to explore such a hypothesis. Such results also highlight the need to optimize an anticoagulation regimen in ECCO_2_R patients. Membrane clotting was more frequently reported with the iLA Activve and led to ECCO_2_R discontinuation in nearly half of the cases. It seems possible that the higher rate of membrane clotting could be explained by easier membrane visualization for the iLA Activve circuit. Biological hemolysis was reported only in patients treated with the Hemolung device. An explanation could be in link with the different configuration of the devices, with different velocity profiles in pumps and membranes. The lack of pressure monitoring could also be involved, since very negative drainage pressures could have been more easily detected with the iLA Activve device. However, the clinical signification of a pure biological hemolysis as defined in the study remains to be established, especially if transient. Nevertheless, there was also a trend to more frequent clinical hemolysis in patients treated with the Hemolung device.

The limits of the study are those of a register, with differences between centers according to indications, learning curves, and frequency of utilization. Indeed, there were no precisely defined criteria for ECCO_2_R initiation, outside the general proposal of initiating ECCO_2_R for achieving ultraprotective ventilation in ARDS patients and to prevent NIV failure or to shorten the duration of intubation in AE-COPD patients. There were a higher number of patients treated with the Hemolung device, which was available prior to the iLA Activve device. Since the choice between devices was not randomized, any comparisons between devices must be considered with caution. Indications outside ARDS, AE-COPD, and asthma were too scarce to infer valuable conclusions (even preliminary) about the efficacy of ECCO_2_R in such settings.

## Conclusion

We report the feasibility of an ECCO_2_R registry in a large metropolis area. Based on the first 70 patients, we report a lower than expected rate of ECCO_2_R device utilization, mainly in severe COPD and ARDS patients. Physiological efficacy was confirmed in these two populations. We also confirmed safety concerns such as hemolysis, bleeding, and thrombosis with different profiles between the devices. Our results could help to design future studies aiming to enhance safety, to demonstrate a still-lacking strong clinical benefit of ECCO_2_R, and to guide the choice between the different devices. In the meantime, use of ECCO_2_R should be limited to clinical trials and/or registries, due to an uncertain benefit-risk ratio.

## Data Availability

Datasets are available in the coordinating center.

## Abbreviations

AE-COPD: Chronic obstructive pulmonary disease acute exacerbation
AE: Adverse event
AGEPS: Assistance Publique–Hôpitaux de Paris Institutional Pharmacy Agency
AP-HP: Assistance Publique–Hôpitaux de Paris
ARDS: Acute respiratory distress syndrome
CO_2_: Carbon dioxide
COPD: Chronic obstructive pulmonary disease
ECCO_2_R: Extracorporeal carbon dioxide removal
ECMO: Extracorporeal membrane oxygenation
ICU: Intensive care unit
IMV: Invasive mechanical ventilation
IQR: Interquartile range
NIV: Non-invasive ventilation
PaCO_2_: Carbon dioxide arterial pressure
PBW: Predicted body weight
PEEP: Positive end-expiratory pressure
RBC: Red blood cells
RCT: Randomized clinical trial
SAE: Serious adverse event
SAPS II: Simplified acute physiology score II
*V*_T_: Tidal volume
Xa: Clotting factor X activated
